# A Case of Adenocarcinoma in a Stoma Site after 27 Years of Stoma Surgery for Hirschsprung’s Disease

**DOI:** 10.70352/scrj.cr.25-0792

**Published:** 2026-07-24

**Authors:** Daisuke Koro, Tatsuya Shonaka, Chikayoshi Tani, Tomohiro Takeda, Masahide Otani, Mizuho Ohara, Toshihiko Hayashi, Yuki Kamikokura, Naoko Aoki, Mishie Tanino, Kimiharu Hasegawa, Hideki Yokoo

**Affiliations:** 1 Department of Surgery, Asahikawa Medical University, Asahikawa, Hokkaido, Japan; 2 Department of Plastic and Reconstructive Surgery, Asahikawa Medical University, Asahikawa, Hokkaido, Japan; 3 Department of Pathology, Asahikawa Medical University Hospital, Asahikawa, Hokkaido, Japan

**Keywords:** adenocarcinoma, surgical stoma, Hirschsprung disease, ileostomy, peristomal neoplasm, late complication, case report

## Abstract

**INTRODUCTION:**

Adenocarcinomas originating at stoma sites are extremely rare. While many cases are associated with colorectal cancer or inflammatory bowel disease, instances without such predisposing factors are even rarer.

**CASE PRESENTATION:**

A 39-year-old man with a history of Hirschsprung’s disease presented with tumor growth at his permanent stoma site, which had been established 27 years earlier. A biopsy confirmed adenocarcinoma. Preoperative imaging, including CT, MRI, and PET-CT, showed no evidence of lymph node or distant metastasis. Immunohistochemical staining (CK7+, CK20+, CDX2+) was consistent with a primary tumor of the small bowel. Based on the preoperative diagnosis of localized disease and the clinical goal of preserving intestinal function, local resection was performed with negative margins. Histopathological examination confirmed a primary ileal adenocarcinoma. The patient remains recurrence-free 30 months postoperatively without adjuvant chemotherapy.

**CONCLUSIONS:**

This report presents a rare case of stoma-site adenocarcinoma arising 27 years after surgery for Hirschsprung’s disease. In long-term survivors of pediatric stoma surgery, chronic physical and chemical irritation may contribute to malignancy even in the absence of a predisposing malignant background. Malignancy at the stoma site can be discovered by patients through self-examination; therefore, both patients and clinicians must recognize the potential risk for early detection.

## Abbreviations

AFP
: alpha-fetoprotein
CA19-9
: carbohydrate antigen 19-9
CDX2
: caudal-type homeobox 2
CEA
: carcinoembryonic antigen
CK7
: cytokeratin 7
CK20
: cytokeratin 20
FAP
: familial adenomatous polyposis
SUV
: standardized uptake value

## INTRODUCTION

Adenocarcinoma at a stoma site is exceedingly rare. Although many cases of stoma site adenocarcinomas are associated with colorectal cancer, FAP, and inflammatory bowel disease, instances devoid of these backgrounds are even rarer.^1)^

We herein report a case of adenocarcinoma at the stoma site 27 years after stoma surgery for Hirschsprung’s disease.

## CASE PRESENTATION

**Fig. 1:**
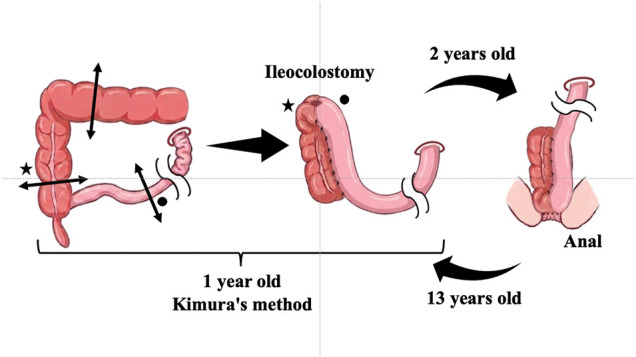
Schema of the prior surgical procedure: Kimura procedure (age 1), radical surgery (age 2), and stoma re-creation (age 13). Double-headed arrows indicate the sites of intestinal transection, while the star and closed dot represent the corresponding regions of the remaining intestine to be anastomosed.

A 39-year-old man presented with a complaint of tumor growth at the stoma site and a history of Hirschsprung’s disease. At 1 year old, a surgical procedure was performed using Kimura’s method. After identifying the intestinal segment containing ganglion cells proximal to the ileocecal region, the anal-side segment and the ileocecal region were resected. The transverse colon was divided at its midpoint, and a side-to-side anastomosis was performed between the pedicled right colon and the distal end of the normal ileum to create an ileocolostomy segment. The anal side of this segment was fashioned into a stoma. The following year, a radical procedure was performed: the aganglionic left colon was resected, and the ileocolostomy segment was pulled through (Soave procedure) into the rectal muscular cuff. At 13 years old, due to anal stenosis, the ileocolostomy segment that had been anastomosed to the anus was elevated as a permanent stoma (**Fig. 1**). The patient had been under the care of the Pediatric Surgery Department.

### Family history

No familial predisposition to gastrointestinal disorders was identified.

### Current medical history

Two years prior to surgery, tumor growth was observed in the transition area between the intestine and the skin at the stoma site. The patient underwent repeated laser ablation at the Department of Dermatology. However, 2 months before surgery, the tumor exhibited rapid progression. A biopsy was performed, and the diagnosis was adenocarcinoma originating from the intestinal tract.

### Clinical examination findings

**Fig. 2:**
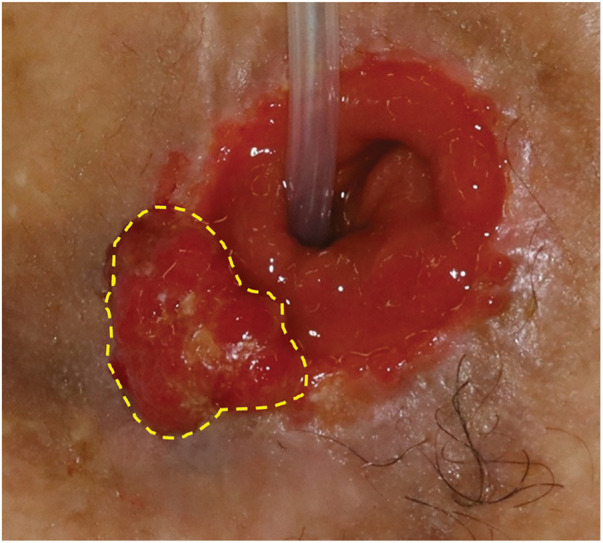
A meticulously defined irregular mass, approximately 2 cm in length, was found in the transition area between the intestine and skin, precisely positioned at 7 o’clock relative to the stoma. The yellow dotted line indicates the extent of the tumor.

The patient’s height and weight were 157 cm and 42 kg, respectively. An irregular mass, measuring approximately 2 cm and well-defined, was observed at the transition area between the intestinal tract and skin at the stoma site (**Fig. 2**).

### Blood and biochemical findings

A complete blood count and biochemical tests revealed no abnormalities. Tumor markers, including CEA (1.3 ng/mL), AFP (2.0 ng/mL), and CA19-9 (13 U/mL), were all within normal limits.

### Imaging findings

**Fig. 3:**
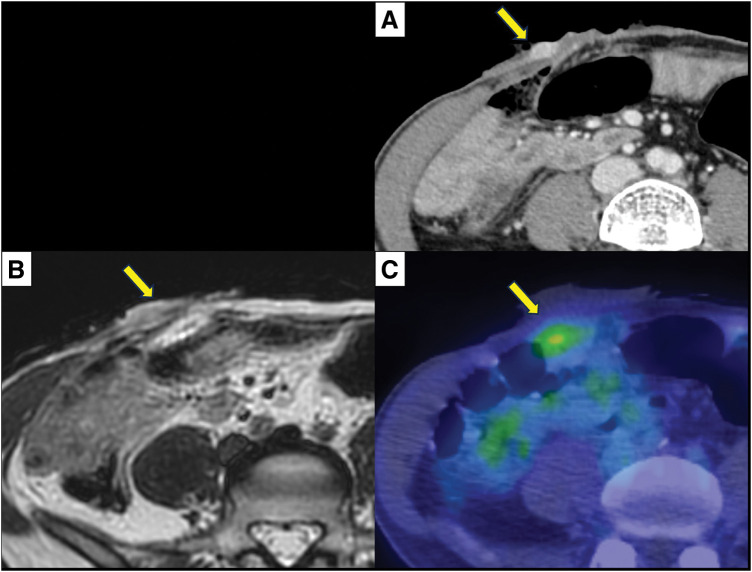
(**A**) Abdominal CT showed irregular, mild thickening of the peristomal skin surface with partial contrast enhancement (arrow). (**B**) Abdominal MRI revealed wall thickening with diffusion restriction at the stoma site without apparent infiltration into the rectus abdominis muscle (arrow). (**C**) FDG-PET/CT demonstrated an accumulation with an SUVmax of 3.0 in the intestinal tract near the stoma site, with no evidence of lymph node or distant metastasis (arrow). SUVmax, maximum standardized uptake value

CT revealed irregular, mild thickening of the perianal skin surface with partial contrast enhancement. No enlarged lymph nodes in the abdominal cavity or distant metastases were observed (**Fig. 3A**). MRI revealed wall thickening with diffusion restriction at the stoma site, with no infiltration of the rectus abdominis muscle (**Fig. 3B**). Fluorodeoxyglucose-PET-CT indicated a maximum SUV of 3.0 in the intestinal tract near the stoma site. However, its exact correspondence to the small lesions identified on CT/MRI remains inconclusive. This discrepancy may be attributed to physiological uptake in the neighboring gastrointestinal tract, which can mask or overlap with signals from small pathological findings. In addition, there was no evidence of abnormal accumulation suggesting lymph node or distant metastasis (**Fig. 3C**).

### Preoperative histopathological findings

Dysplasia of the cells forming the luminal structures was evident and was characterized by irregularly sized nuclei and numerous mitotic figures. Positive immunostaining for CK7, CK20, and CDX2 is consistent with a primary tumor of the small bowel.

### Preoperative diagnosis

Given the absence of any abnormal accumulation outside the tumor area on PET-CT, macroscopic and imaging evaluations indicated that the tumor invasion was limited to the mucosa. And, given the goal of preserving the colon to maintain intestinal water absorption capacity, we decided to perform not bowel resection but local resection.

### Surgical findings

**Fig. 4:**
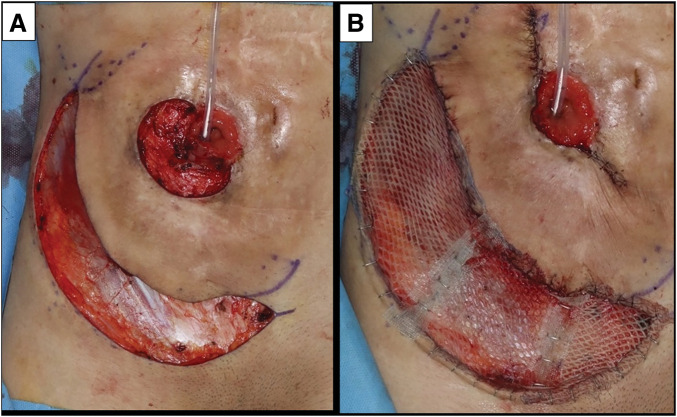
Surgical findings. (**A**) The tumor was meticulously excised with a margin of approximately 1 cm on the skin side and 5 mm on the intestinal side, precisely located at 7 o’clock relative to the stoma. A skin flap was created to mobilize the colostomy skin. (**B**) The ensuing skin defect underwent plastic surgery, utilizing skin flap surgery and bilobed skin grafting sourced from the left thigh.

Tumor resection with a 1-cm margin on the skin side and a 5-mm margin on the intestinal side was performed at the 7 o’clock position during colostomy (**Fig. 4A**). Plastic surgery of the left thigh with skin flap surgery and segmental skin grafting addressed the skin defect (**Fig. 4B**). The operation lasted for 2 h and 23 min with minimal blood loss (32 mL).

### Excised specimen

**Fig. 5:**
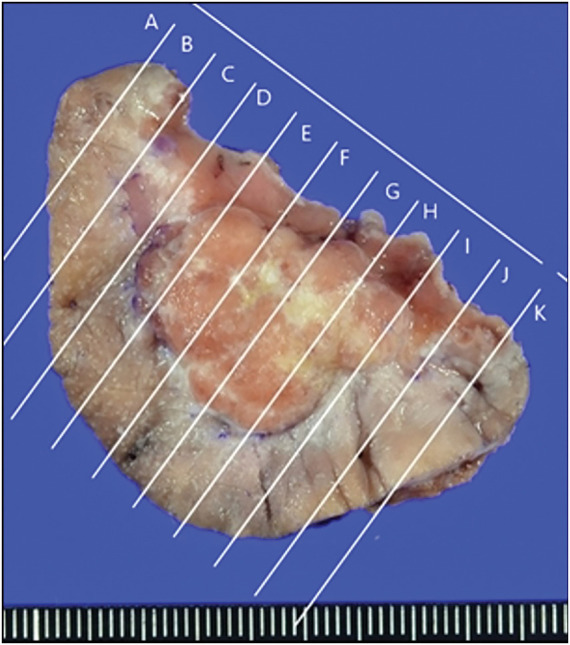
Excised specimen. The excised specimen, measuring 52 × 35 × 13 mm, exhibited a well-defined, light-brown, substantial mass measuring 24 × 16 × 9 mm at the transition area between the mucosa and skin of the stoma.

The excised specimen measured 52 × 35 × 13 mm, revealing a well-defined, light-brown, substantial mass of 24 × 16 × 9 mm at the mucosa–dermis transition area of the stoma site. The resection margins were negative (**Fig. 5**).

### Histopathological findings

**Fig. 6:**
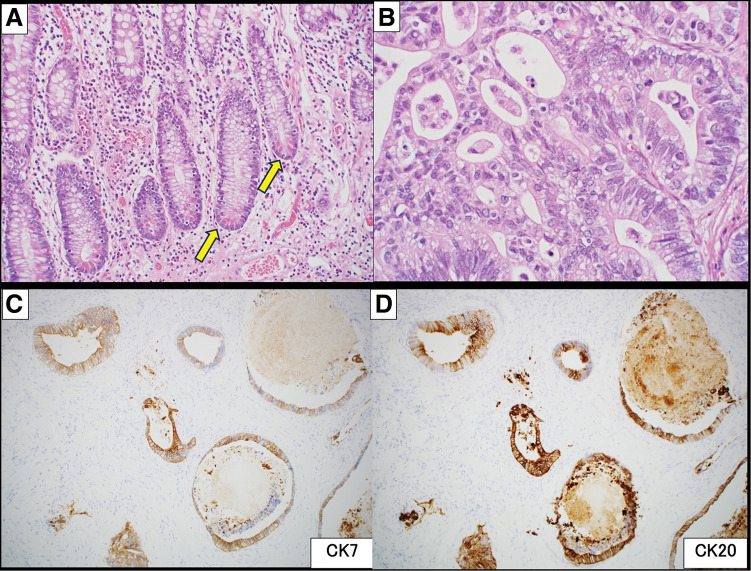
Excision pathology. (**A**) HE non-tumor area (x200); (**B**) HE tumor area (x400); (**C**) CK7 (x100); (**D**) CK20 (x100). (**A**) Paneth cells (arrows) were identified in the intestinal mucosa of the non-tumor area, a histological characteristic consistent with a primary ileal origin. (**B**) In the tumor area, proliferating atypical cells demonstrated features such as nuclear enlargement, irregular size of nuclei, and chromatin hyperplasia. Positive immunostaining for CK7. (**C**) and CK20 (**D**) aligned with a primary tumor of the digestive system.

The mass exhibited infiltrating and proliferating atypical cells with enlarged nuclei, irregular nuclear sizes, and increased chromatin. Distinct fused tubular structures and partially indistinct lumens with irregular meandering were observed. Paneth cells in the intestinal mucosa of the non-neoplastic area indicated a primary ileal tumor (**Fig. 6A** and **6B**). Positive immunostaining for CK7 and CK20 aligned with the primary tumor of the digestive system (**Fig. 6C** and **6D**). In the intestinal segment continuous with the skin, the tumor invades beyond the muscularis propria; however, the tissue beneath it consists of subcutaneous adipose tissue. For this reason, whether the standard TNM staging is applicable here is debatable; however, if classified, the tumor is estimated to be T3, NX, M0, Stage IIa. The resection margins were negative, and the other pathological findings were type 5, 16 mm, tub2>tub1>por2, Ly0, V1b, BD1, pNX, R0, and CurA.

### Postoperative course

The patient’s recovery was uneventful, and discharge occurred on POD 18 after ensuring proper skin graft integration while managing the stoma. Although vascular invasion was identified, we decided to opt for strict follow-up without performing additional radical resection. This is because we carefully considered the aforementioned risks, and the patient did not wish to undergo the procedure. Adjuvant chemotherapy was not administered, and the patient remained alive and recurrence-free 30 months postoperatively.

## DISCUSSION

The emergence of cancer at the stoma site represents a rare and late complication of stoma surgery.^2)^ While most peristomal cancers are associated with underlying conditions, such as colorectal cancer, familial adenomatous polyposis, or inflammatory bowel disease,^3)^ our patient exhibited no predisposing matrix for malignancy.

This case is particularly uncommon, as it involves a malignant tumor at the stoma–skin interface. Factors potentially contributing to the development of colostomy cancer in patients lacking a predisposition to malignancy include physical irritation from braces and skin protectants, chemical irritation from alkaline stools in the ileum, colonic epithelialization, and inflammatory bowel disease.^4)^ There are recommendations for periodic screening of colostomies at 20 years post-colostomy or at ≥50 years of age for cases of FAP, and at 25 years post-colostomy or at ≥60 years of age for inflammatory bowel disease.^5)^ However, this case is distinctive, as it involves cancer without a background of inflammatory bowel disease or malignancy, suggesting a possible etiology rooted in physical and chemical irritation from braces, skin protectants, and alkaline stools in the ileum. In addition, there is a possibility that repeated laser ablation performed on the adenoma induced the adenoma–carcinoma sequence.

Malignant tumors at the ileostomy are infrequent, and we compiled the existing literature on this subject. Among the cases of ileostomy cancer identified in the PubMed database between 2002 and 2022, there were 6 cases that underwent surgical resection and for which detailed clinical information could be confirmed^6–11)^ (**Table 1**). The median age of the patients was 65 (range: 37–85) years, with a median time from stoma construction to cancer development of 258 (range: 120–468) months; more than 20 years had passed since surgery in 3 cases. In all cases, the stoma type was an end ileostomy. The background diseases included inflammatory bowel disease in 4 cases and FAP in 2. There were no cases of ileostomy cancer arising in patients with a background of Hirschsprung’s disease. This summary underscores the exceptional rarity of patients with a background of Hirschsprung’s disease, as exemplified by the present case, necessitating further case accumulation for a comprehensive understanding.

**Table 1:** Summary of data in previous reports

Variables	Values
Patient characteristics	
Age (years), median (min–max)	65 (37–85)
Gender (male:female)	1:5
Age of stoma (month), median (min–max)	
Ileostomy	258 (120–468)
Indication for stoma creation	
Inflammatory bowel disease	4
Familial adenomatous polyposis	2
Surgical procedures	
Stoma reconstruction including bowel resection	5
Tumor resection	1

The time between stoma creation and the cancer onset was analyzed, revealing a median of 258 (range: 120–468) months for ileostomies, with the latter consistently exhibiting a longer duration in all cases, surpassing 10 years after ileostomy creation.

The literature lacks standardized methods for the selection of surgical approaches. Reconstruction of the stoma is often performed. However, there is a report that even for cancer at the stoma site, local excision was possible if it was early-stage cancer limited to the mucosa. Depending on the depth of tumor invasion and preoperative diagnosis, lymph node dissection may be omitted. In our case, intestinal resection and lymph node dissection were omitted, prioritizing consideration of the QOL and other factors. However, rigorous follow-up is imperative to monitor patients for potential recurrence.

## CONCLUSIONS

In conclusion, this report presents a singular case of adenocarcinoma at the stoma–skin interface arising 27 years after stoma creation, in the context of Hirschsprung’s disease. Malignancy at the stoma site can be discovered by patients through self-examination; therefore, both patients and clinicians must recognize the potential risk for early detection.

## DECLARATIONS

### Availability of data and materials

The datasets supporting the conclusions of this article are included within the article.
